# Animated Biofeedback for Pediatric Functional Voiding Dysfunction: A Systematic Review and Meta-Analysis of Postvoid Residual Outcomes

**DOI:** 10.7759/cureus.111713

**Published:** 2026-06-29

**Authors:** Hafizar Hafizar, Irfan Wahyudi, Putu Angga Risky Raharja, Gerhard Reinaldi Situmorang, Arry Rodjani

**Affiliations:** 1 Department of Urology, Rumah Sakit Umum Pusat Nasional Dr. Cipto Mangunkusumo, Faculty of Medicine, Universitas Indonesia, Jakarta Pusat, IDN

**Keywords:** animated biofeedback, dysfunctional voiding, gamified biofeedback, pediatric voiding dysfunction, postvoid residual

## Abstract

Animated or gamified biofeedback has been introduced to improve engagement during pelvic floor retraining in children with functional voiding dysfunction. However, its incremental clinical benefit beyond standard urotherapy or conventional biofeedback remains uncertain. This systematic review aimed to synthesize available evidence on animated or gamified biofeedback for pediatric nonneurogenic functional voiding dysfunction, with exploratory quantitative analysis of postvoid residual (PVR) urine volume. A systematic search of PubMed, Cochrane Library, and ScienceDirect was conducted in accordance with Preferred Reporting Items for Systematic Reviews and Meta-Analyses 2020 guidance. Comparative studies evaluating animated or gamified biofeedback in children with dysfunctional voiding, dysfunctional elimination syndrome, or nonneuropathic underactive bladder were included. PVR urine volume was pooled using a random-effects model when extractable data were available. Other outcomes were summarized narratively.

Three comparative studies involving 170 children were included. Two studies compared animated biofeedback plus standard urotherapy with urotherapy alone, whereas one compared animated with nonanimated biofeedback. Exploratory meta-analysis showed no statistically significant reduction in PVR urine volume with animated or gamified biofeedback compared with control regimens (MD, -6.83 mL; 95% CI, -29.27 to 15.62; p = 0.55), with substantial heterogeneity (I² = 89%). Narrative findings suggested more favorable outcomes when biofeedback was added to urotherapy, but the direct comparison did not demonstrate clear superiority of animation over conventional biofeedback. The available evidence is limited by small sample size, heterogeneous diagnoses, inconsistent comparator structures, and imprecision. The pooled estimate should therefore be interpreted as exploratory rather than confirmatory. Current evidence is insufficient to confirm that animation or gamification provides additional therapeutic benefit beyond conventional biofeedback. Larger standardized comparative studies are needed.

## Introduction and background

Functional lower urinary tract dysfunction remains one of the most frequently encountered conditions in pediatric urology, accounting for a substantial proportion of outpatient referrals worldwide [1]. Among these disorders, dysfunctional voiding (DV) and nonneuropathic underactive bladder (UAB) are particularly prevalent and clinically challenging. DV is characterized by inappropriate contraction of the external urethral sphincter or pelvic floor musculature during the voiding phase in neurologically intact children, leading to discoordinated bladder emptying [2]. Similarly, UAB in children involves insufficient detrusor contractility, often accompanied by compensatory behavioral patterns that further impair effective voiding [3]. Although these entities differ mechanistically, both share a common endpoint: inefficient bladder emptying and persistence of lower urinary tract symptoms.

The clinical consequences of untreated or inadequately treated voiding dysfunction extend beyond simple urinary frequency or incontinence. Children with DV or UAB frequently present with recurrent urinary tract infections, elevated postvoid residual (PVR) urine volume, vesicoureteral reflux, and, in some cases, progressive upper tract deterioration [3]. Additionally, chronic voiding dysfunction can negatively affect psychosocial development. Children may experience embarrassment, reduced self-esteem, social withdrawal, and school-related difficulties due to persistent urinary symptoms [4]. These multidimensional impacts underscore the importance of early, effective, and sustainable treatment strategies.

The pathophysiological foundation of DV lies in learned maladaptive voiding behavior rather than structural abnormality [2]. Persistent contraction of the pelvic floor during voiding produces characteristic staccato or interrupted uroflow patterns and increases outlet resistance. Over time, this may lead to secondary detrusor instability or reduced compliance. In UAB, insufficient detrusor contraction results in prolonged voiding time and significant residual urine, predisposing to infection and bladder dysfunction [3]. Objective evaluation typically includes uroflowmetry combined with surface electromyography (EMG) to detect inappropriate sphincter activation, as well as ultrasound measurement of PVR volume [1]. These tools provide reproducible parameters for both diagnosis and monitoring of treatment response.

Standard urotherapy is widely accepted as first-line management for pediatric DV and related functional voiding disorders [2]. This approach emphasizes nonpharmacological measures such as timed voiding, optimizing hydration, managing constipation, toilet posture education, and behavioral reinforcement. While many children respond favorably to structured urotherapy, a significant subset exhibits persistent abnormal uroflow patterns, elevated PVR, or ongoing incontinence despite adequate adherence [5]. In such cases, escalation of therapy becomes necessary.

Pelvic floor biofeedback therapy was introduced as a targeted neuromuscular retraining technique to address the core discoordination underlying DV [5]. By providing real-time visual feedback of pelvic floor EMG activity, biofeedback enables children to recognize inappropriate muscle contraction and learn voluntary relaxation during the voiding phase. Multiple prospective studies have demonstrated that biofeedback can improve uroflowmetry parameters, reduce residual urine volume, and alleviate symptoms in children with treatment-resistant voiding dysfunction [5,6]. These improvements are thought to arise from reinforcement of correct motor patterns and restoration of coordinated detrusor-sphincter interaction.

To further enhance engagement and adherence, animated or gamified biofeedback systems were developed, particularly for younger children [6]. These systems incorporate interactive visual elements, such as moving graphics, game-based scoring, or animated characters, to maintain attention during sessions and provide immediate, intuitive reinforcement of correct pelvic floor relaxation. From a motor learning perspective, gamification may strengthen cognitive-motor integration and accelerate skill acquisition [7]. Early reports suggested that animated biofeedback could yield rapid improvements in both subjective symptoms and objective measures, potentially surpassing conventional nonanimated approaches [8].

However, evidence regarding the incremental benefit of animation remains inconsistent. Some trials report superior outcomes with animated systems compared with urotherapy alone, while direct comparisons between animated and nonanimated biofeedback often demonstrate comparable success rates [9]. Furthermore, heterogeneity in study design, diagnostic categories, session frequency, and outcome definitions complicates the interpretation of individual findings. Although previous systematic reviews have examined biofeedback therapy in children with nonneuropathic voiding disorders, they did not specifically isolate animated or gamified modalities as a distinct intervention category [7]. Consequently, whether animation itself confers additional measurable clinical benefit beyond standard biofeedback or conservative urotherapy remains unclear [6-9].

Given the increasing integration of digital and gamified rehabilitation technologies into pediatric clinical practice, clarifying their true therapeutic value is essential. Animated biofeedback systems may require additional resources, equipment, and training; thus, evidence-based justification for their use is necessary to inform clinical guidelines and optimize healthcare allocation.

Despite this clinical rationale, evaluating animated or gamified biofeedback as a distinct therapeutic modality is methodologically challenging. Pediatric functional voiding dysfunction is not a single homogeneous entity, and conditions such as DV and nonneuropathic underactive bladder differ in their dominant pathophysiological mechanisms, despite sharing incomplete bladder emptying as a common clinical endpoint. Similarly, existing trials have used different comparator structures: some assess animated biofeedback as an add-on to standard urotherapy, whereas others compare animated interfaces directly with conventional nonanimated biofeedback. These distinctions are important because the first comparison evaluates the overall effect of adding biofeedback-based neuromuscular retraining to conservative care, while the second evaluates the incremental value of animation or gamification beyond biofeedback itself. Therefore, any quantitative synthesis in this field must be interpreted cautiously, particularly when the number of randomized trials is small and clinical heterogeneity is substantial.

Therefore, the present systematic review aimed to synthesize the available evidence on animated or gamified pelvic floor biofeedback for children and adolescents with functional voiding dysfunction. The primary focus was to clarify whether animated biofeedback provides measurable clinical or physiological benefit compared with standard urotherapy or conventional nonanimated biofeedback. Because of anticipated heterogeneity in diagnosis, comparator structure, and outcome reporting, PVR urine volume was analyzed quantitatively where data were sufficiently extractable, while other outcomes were summarized narratively. The quantitative synthesis was considered exploratory and was intended to support, rather than replace, clinically contextualized interpretation of the available evidence.

## Review

Methods

This systematic review and meta-analysis has been registered in the Prospective Register of Systematic Reviews (PROSPERO) database with registration number CRD420261326418. This review followed the registered PROSPERO protocol. The protocol included several clinically relevant voiding-related outcomes, including PVR urine volume, uroflowmetry parameters, symptom scores, treatment success, urinary tract infection frequency, recurrence, and adverse events. However, quantitative pooling was restricted to PVR urine volume because this was the only outcome reported with sufficient numerical consistency across the included comparative studies. This was considered an analytical refinement from the registered protocol rather than a substantive protocol deviation, as no outcome was added post hoc and all other prespecified outcomes were retained and summarized narratively. No other deviations from the registered protocol occurred.

Study Design and Reporting

This study was designed as a systematic review with an exploratory meta-analysis and was conducted in accordance with the Preferred Reporting Items for Systematic Reviews and Meta-Analyses (PRISMA) 2020 guidelines. The review aimed to synthesize available comparative evidence on animated or gamified pelvic floor biofeedback for pediatric nonneurogenic functional voiding dysfunction. Because of the limited number of eligible studies and anticipated heterogeneity in diagnosis, comparator structure, and outcome reporting, quantitative pooling was restricted to PVR urine volume when extractable data were available. Other clinically relevant outcomes were summarized narratively to preserve clinical interpretability.

Eligibility Criteria

Studies were eligible for inclusion if they met the following criteria: 1) involved a pediatric population aged 18 years or younger with nonneurogenic functional voiding dysfunction, including DV, dysfunctional elimination syndrome, or nonneuropathic UAB; 2) evaluated animated, gamified, computer-assisted, or visual EMG-based pelvic floor biofeedback as the main intervention; 3) included a comparator group receiving either standard urotherapy/conservative management without biofeedback or conventional nonanimated biofeedback; and 4) reported at least one clinically relevant outcome related to voiding function, such as PVR urine volume, uroflowmetry parameters, symptom scores, treatment success, urinary tract infection frequency, or recurrence.

For the quantitative synthesis, studies were included only if they reported extractable PVR urine volume data as mean and standard deviation, or provided sufficient information to derive these values. Because the available literature included different comparator structures, studies comparing animated biofeedback with urotherapy alone and studies comparing animated biofeedback with nonanimated biofeedback were considered separately in the clinical interpretation. The pooled analysis of PVR urine volume was therefore regarded as exploratory rather than confirmatory.

Studies were excluded if they involved patients with neurogenic bladder, congenital or structural lower urinary tract abnormalities, spinal cord pathology, or other anatomical conditions directly explaining voiding dysfunction. Adult studies, case reports, review articles, conference abstracts without sufficient extractable data, noncomparative reports, and studies without relevant clinical or physiological outcomes were also excluded. Studies were not excluded solely on the basis of observational design for the qualitative synthesis; however, only comparative studies with extractable PVR urine data were eligible for quantitative pooling.

Information Sources and Search Strategy

A systematic literature search was conducted using PubMed, Cochrane Library, and ScienceDirect to identify randomized studies evaluating animated or gamified biofeedback for pediatric voiding dysfunction. The final database search was conducted on February 1, 2026. Searches were performed from database inception to the final search date. No language restriction was applied/only English-language full-text articles were included. No restriction was applied based on publication year. PubMed was selected to capture biomedical and pediatric urology literature, the Cochrane Library was searched to identify controlled trials and systematic evidence, and ScienceDirect was included to broaden the retrieval of full-text clinical and rehabilitation-related studies. However, the search was limited to these three databases. Embase, Scopus, and Web of Science were not searched. Therefore, although the principal randomized comparative studies in this narrow field were identified through the databases searched, the possibility that additional relevant records were missed cannot be excluded. The search strategy was developed using three core concepts: pediatric functional voiding disorders, biofeedback-based intervention, and the child/adolescent population. The final database-specific search strings were as follows:

PubMed: ("dysfunctional voiding" OR "voiding dysfunction" OR "underactive bladder" OR "dysfunctional elimination syndrome") AND ("animated biofeedback" OR "biofeedback" OR "pelvic floor biofeedback" OR "computer-assisted biofeedback") AND (child OR children OR pediatric OR paediatric OR adolescent)

Cochrane Library: ("dysfunctional voiding" OR "voiding dysfunction" OR "underactive bladder" OR "dysfunctional elimination syndrome") AND ("animated biofeedback" OR "biofeedback" OR "pelvic floor biofeedback") AND (children OR pediatric OR adolescent)

ScienceDirect: ("dysfunctional voiding" OR "underactive bladder") AND ("animated biofeedback" OR biofeedback) AND (children OR pediatric)

The ScienceDirect search string was intentionally simplified to improve retrieval precision while remaining within the platform’s practical Boolean limitations. Retrieved records were screened by title and abstract, followed by full-text assessment according to the predefined eligibility criteria.

Study Selection

All retrieved records were imported into a reference management file, and duplicate records were removed before screening. Titles and abstracts were screened independently by five reviewers according to the predefined eligibility criteria. Records considered potentially eligible by either reviewer were retrieved for full-text assessment. Full-text eligibility was then assessed independently by five reviewers. Disagreements at both screening and full-text stages were resolved through discussion and consensus, with involvement of a sixth reviewer when required.

Full-text assessment was then performed using the predefined eligibility criteria. Studies were included in the qualitative synthesis if they evaluated animated or gamified biofeedback in pediatric nonneurogenic functional voiding dysfunction and reported relevant clinical or physiological outcomes. Studies were included in the exploratory quantitative synthesis only when PVR urine volume data were reported in an extractable format. Reasons for exclusion at the full-text stage were documented. The overall selection process was summarized using the PRISMA 2020 flow diagram.

Data Extraction

Data were extracted using a standardized extraction form. The extracted variables included first author, publication year, country, study design, sample size, participant age, diagnostic category, inclusion criteria, intervention characteristics, comparator structure, session frequency, treatment duration, follow-up duration, outcome definitions, and main findings.

For intervention characteristics, data were collected on the type of animated or gamified biofeedback system, use of EMG-based feedback, integration with pelvic floor muscle exercises, and cointerventions such as standard urotherapy. Comparator groups were categorized as either standard urotherapy/conservative management without biofeedback or conventional nonanimated biofeedback. This distinction was recorded because it directly affects the interpretation of whether the observed effect reflects biofeedback itself or the additional value of animation.

For quantitative synthesis, PVR urine volume data were extracted as mean, standard deviation, and sample size for each group at the latest available follow-up. When studies reported multiple timepoints, the longest follow-up after completion of treatment was prioritized. If data were insufficient for pooling, the outcome was retained for narrative synthesis only. Other outcomes, including uroflowmetry parameters, symptom scores, clinical success, recurrence, urinary tract infection frequency, and adverse events, were extracted descriptively.

Outcome

The outcomes of interest were selected to capture both physiological and clinical aspects of pediatric functional voiding dysfunction. The main outcome for quantitative synthesis was PVR urine volume (PVR), expressed in milliliters, because it was the most consistently reported objective parameter across the included comparative studies and directly reflects bladder emptying efficiency.

Other outcomes of interest included uroflowmetry parameters, such as maximum urinary flow rate, voiding time, voided volume, and uroflow pattern normalization; pelvic floor EMG activity during voiding; symptom scores, including validated voiding dysfunction symptom scores when available; clinical treatment success; urinary tract infection frequency; recurrence; and adverse events. These outcomes were summarized narratively because reporting methods, definitions, follow-up timepoints, and statistical formats varied substantially across studies, preventing reliable quantitative pooling.

Treatment success was defined according to the criteria used in each individual study, including complete or partial symptom improvement, normalization of uroflowmetry or EMG findings, reduction in PVR, or combined clinical and objective response. Because these definitions were not standardized across trials, treatment success was not pooled quantitatively.

Therefore, PVR was treated as the only outcome eligible for exploratory meta-analysis, while symptom response, uroflowmetry improvement, clinical success, recurrence, and safety outcomes were interpreted descriptively within the systematic review framework. This distinction was made to avoid overestimating the strength of evidence from heterogeneous and inconsistently reported endpoints.

Risk of Bias Assessment

Risk of bias was assessed using the Cochrane Risk of Bias 2 (RoB 2) tool for randomized trials. The following domains were evaluated: bias arising from the randomization process, bias due to deviations from intended interventions, bias due to missing outcome data, bias in the measurement of the outcome, and bias in the selection of the reported result.

Judgments were made at the domain level and categorized as low risk of bias, some concerns, or high risk of bias according to RoB 2 guidance. Because biofeedback-based behavioral interventions cannot feasibly blind participants or therapists, the lack of blinding was carefully considered when judging deviations from intended interventions and outcome measurement. Objective outcomes, such as PVR and uroflowmetry parameters, were considered less vulnerable to subjective measurement bias than symptom-based outcomes, although incomplete reporting of assessor blinding was still noted.

An overall risk-of-bias judgment was assigned to each study based on the domain-level assessments. Studies were not automatically classified as low risk solely because they were randomized. When allocation concealment, assessor blinding, or selective reporting could not be clearly determined, the study was judged to have some concerns. A domain-by-domain risk-of-bias summary was prepared to improve transparency of the assessment.

Certainty of Evidence

The certainty of evidence for the exploratory pooled outcome was assessed narratively using principles adapted from the Grading of Recommendations Assessment, Development and Evaluation framework. Certainty was considered in relation to risk of bias, inconsistency, indirectness, imprecision, and publication bias. Because the quantitative synthesis included only three small studies with substantial clinical and statistical heterogeneity, the certainty of the evidence for the pooled PVR outcome was interpreted with caution.

Statistical Analysis

Quantitative synthesis was performed for PVR urine volume because it was the only outcome reported with sufficient numerical consistency across the included comparative studies. PVR was analyzed as a continuous outcome and expressed as mean difference (MD) with 95% confidence intervals (CI). When posttreatment PVR values were available, the final follow-up values were prioritized to reflect residual bladder emptying status after completion of the intervention.

Given the anticipated clinical and methodological heterogeneity across studies, a random-effects model was used for all pooled analyses. Heterogeneity was assessed using the I² statistic and Cochran’s Q test. I² values of approximately 25%, 50%, and 75% were interpreted as low, moderate, and high heterogeneity, respectively. Because the included studies differed in their diagnoses and comparator structures, the pooled estimate was interpreted as exploratory rather than confirmatory.

Clinical heterogeneity was assessed qualitatively by examining differences in patient diagnoses, intervention protocols, session frequencies, follow-up durations, and comparator groups. In particular, studies comparing animated biofeedback with standard urotherapy alone were distinguished from the study comparing animated biofeedback with conventional nonanimated biofeedback. Because only three studies were available, formal subgroup meta-analysis was not considered statistically reliable. Instead, the comparator structure was addressed through narrative stratification in the Results and Discussion sections.

To explore the robustness of the pooled estimate, a leave-one-out sensitivity analysis was planned by sequentially excluding each study and observing the direction and magnitude of the pooled effect. However, given the very small number of studies, this analysis was interpreted with caution and used only to assess whether the pooled result was disproportionately influenced by any single trial. A prediction interval was also considered to illustrate the expected range of effects in future comparable settings, but its interpretation was limited by the small number of studies and substantial between-study variability.

Assessment of publication bias using funnel plot asymmetry or formal regression-based tests was not performed because fewer than 10 studies were included, making such analyses statistically underpowered and potentially misleading. All pooled analyses were performed using Review Manager version 5.4 (The Cochrane Collaboration, London, UK). PVR urine volume was analyzed as a continuous outcome using a random-effects model and expressed as MD with 95% CIs. Heterogeneity was assessed using the I² statistic and Cochran’s Q test within Review Manager. The forest plot was generated using Review Manager version 5.4. R software (R Foundation for Statistical Computing, Vienna, Austria) was not used in the final analysis. A two-sided p value of <0.05 was considered statistically significant.

Results

Study Selection

The systematic database search identified 202 records, comprising 142 records from PubMed, 12 from the Cochrane Library, and 48 from ScienceDirect. One duplicate record was removed before screening, leaving 201 records for title and abstract screening. Of these, 197 records were excluded because they were not aligned with the review question, did not evaluate animated or gamified biofeedback, did not involve pediatric functional voiding dysfunction, or did not report relevant clinical outcomes.

**Figure 1:**
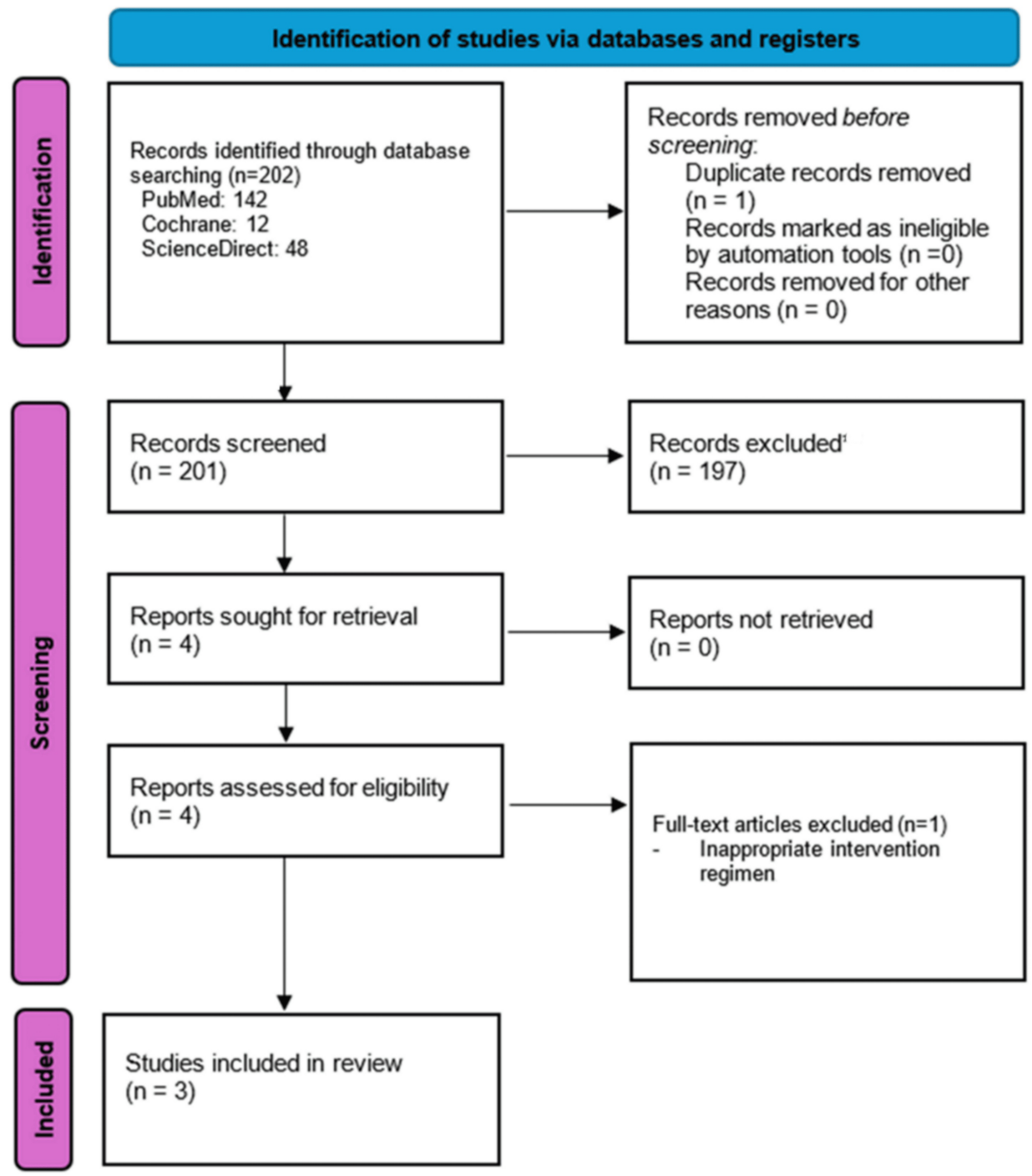
Diagram flow of literature search strategy for this meta-analysis

Four full-text articles were sought and assessed for eligibility. One article was excluded because the intervention/comparator regimen was not appropriate for the predefined review framework. Ultimately, three studies met the eligibility criteria and were included in the qualitative synthesis and exploratory quantitative synthesis. The selection process is summarized in the PRISMA 2020 flow diagram (Figure 1).

Study Characteristics

Three prospective randomized comparative trials involving 170 pediatric participants were included in the analysis. Sample sizes ranged from 40 to 80 participants per study. The trials were conducted in Iran (two studies) and Turkey (one study), reflecting a relatively homogeneous geographic distribution but potentially similar clinical practice environments (Table 1).

**Table 1:** Characteristics and results of the included studies UAB: underactive bladder; DV: dysfunctional voiding; DUI: daytime urinary incontinence; RCT: randomized controlled trial; DES: dysfunctional elimination syndrome; EMG: electromyography; DVSS: Dysfunctional Voiding Scoring System; PVR: postvoid residual; UF-EMG: uroflowmetry paired with electromyography; UTI: urinary tract infection

Study	Country	Study design	Population (age range)	Diagnosis (DV/DUI/UAB)	Sample size (I/C)	Intervention (type of gamified biofeedback)	Comparator (standard urotherapy)	Session frequency and duration	Outcome definition for success	Follow-up duration	Main findings	Risk of bias
Kajbafzadeh et al. [10]	Iran	RCT	Mean 8.5 ± 2.7 years (I); 9.0 ± 2.3 years (C); range 5-16 years	Dysfunctional voiding within DES	40/40	Animated computer-game-based EMG biofeedback + pelvic floor exercises + behavioral modification	Behavioral modification only (timed voiding, hydration, high-fiber diet)	Twice weekly; min 6 to max 12 sessions (mean 9.6 ± 1.3)	Clinical improvement based on DVSS reduction and resolution of subjective/objective voiding abnormalities	6 and 12 months	Greater DVSS reduction, normalization of uroflow patterns, ↓PVR, ↓voiding time, ↑max/avg flow; superior to control	Low
Ladi-Seyedian et al. [8]	Iran	Double-blind RCT	Mean 8.9 ± 2.3 years (I); 9.1 ± 2.7 years (C); range 5-16 years	Nonneuropathic UAB	25/25 (one dropout in C)	Animated computer-game EMG biofeedback + pelvic floor muscle exercises + standard urotherapy	Standard urotherapy only (hydration, timed voiding, posture, diet)	Weekly; min 10 to max 15 sessions	Improvement in subjective (voiding frequency, continence) and objective (UF-EMG normalization, ↓PVR) outcomes	6 and 12 months	Greater ↑voiding frequency, ↓PVR, ↓voiding time, ↑Qmax; fewer UTIs vs. control	Low
Oktar et al. [9]	Turkey	Prospective randomized comparative study	Mean 9.5 ± 3.6 years (animated); 10.5 ± 3.2 years (nonanimated)	DV	20/20	Animated EMG-based biofeedback (computer animations) + standard urotherapy	Nonanimated EMG biofeedback + standard urotherapy	Weekly; mean 5.2 ± 1.9 sessions	Clinical success = EMG cessation + symptom resolution + uroflow improvement	≥1 year (recurrence assessed)	No difference in clinical success between animated vs. nonanimated biofeedback	Some concerns

Participants were children and adolescents aged approximately 5-16 years diagnosed with functional lower urinary tract disorders, including DV, dysfunctional elimination syndrome (DES), and nonneuropathic UAB. All included populations were neurologically intact, and patients with anatomical abnormalities or neurogenic bladder conditions were excluded from the primary trials, ensuring clinical homogeneity within the functional voiding spectrum.

Across studies, the intervention consisted of EMG-based pelvic floor biofeedback delivered via animated or gamified computer-assisted interfaces. The animated systems typically displayed visual graphics or game-based feedback to encourage active participation and motor learning. Biofeedback sessions were combined with pelvic floor muscle exercises and standard urotherapy measures, including adequate hydration, timed voiding schedules, toilet posture training, constipation management, and dietary guidance.

Comparator groups differed across studies. Two trials compared animated/gamified biofeedback plus standard urotherapy against standard urotherapy alone. One trial directly compared animated biofeedback with nonanimated biofeedback, with both arms receiving the same urotherapy background care. This difference in comparator structure is clinically relevant and likely contributed to heterogeneity in pooled analyses.

Treatment intensity and duration varied. One study administered sessions twice weekly, while others used weekly sessions. The number of sessions ranged from approximately 5 to 15, depending on protocol and clinical response. Follow-up duration ranged from 6 to 12 months in two studies, with one study extending follow-up beyond one year and reporting recurrence patterns requiring maintenance therapy in a subset of participants.

The included studies were clinically and methodologically heterogeneous from the outset. Two studies enrolled children with DV or dysfunctional elimination syndrome, whereas one study focused on nonneuropathic UAB. Although these diagnoses belong to the broader spectrum of pediatric nonneurogenic functional voiding dysfunction, they differ in their dominant pathophysiological mechanisms and may not respond identically to pelvic floor retraining. Comparator structures also differed across studies. Two trials evaluated animated biofeedback as an adjunct to standard urotherapy and compared it with urotherapy alone, whereas one trial directly compared animated biofeedback with conventional nonanimated biofeedback, with both groups receiving background urotherapy. Therefore, the available evidence addressed two related but distinct clinical questions: whether biofeedback adds benefit to urotherapy, and whether animation adds benefit beyond biofeedback itself.

Risk of Bias Within Studies

**Figure 2:**
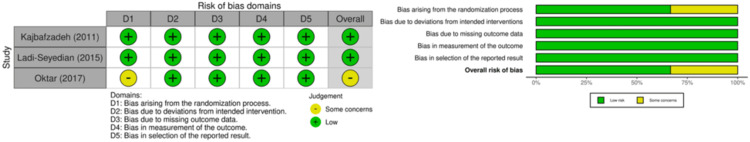
Risk of bias assessment result of the included studies Source: [8-10]

The overall methodological quality of the included studies was generally acceptable, although some concerns were identified in one trial. Based on the RoB 2 assessment, Ladi-Seyedian et al. and Kajbafzadeh et al. were judged to have low risk of bias across all five assessed domains, including the randomization process, deviations from intended interventions, missing outcome data, outcome measurement, and selection of the reported result. Accordingly, both studies were classified as having an overall low risk of bias (Figure 2).

Oktar et al. were judged to have some concerns in the domain of the randomization process, mainly because the reporting of random sequence generation and allocation concealment was less complete. However, the remaining domains were considered low risk, including deviations from intended interventions, missing outcome data, measurement of outcomes, and selection of reported results. The overall risk of bias for this study was therefore categorized as some concerns rather than low risk.

Across the included evidence, most domain-level judgments were classified as low risk. Nevertheless, the presence of some concerns in the randomization process of one included study, combined with the small number of trials and differences in comparator structure, supports a cautious interpretation of the pooled estimate. These limitations do not invalidate the included evidence but reduce confidence in drawing definitive conclusions regarding the incremental effect of animated or gamified biofeedback.

Effects of Interventions

**Figure 3:**
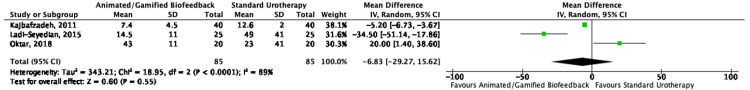
Exploratory pooled analysis of post-void residual urine volume across studies with heterogeneous diagnoses and comparator structures SD: standard deviation; CI: confidence interval; IV: inverse variance Source: [8-10]

PVR volume: PVR urine volume was the only outcome reported with sufficient numerical consistency to permit exploratory quantitative synthesis. All three included studies reported PVR outcomes; however, they differed substantially in their diagnoses and comparator structures. Two studies evaluated animated biofeedback added to standard urotherapy and compared it with urotherapy alone, whereas one study compared animated biofeedback with conventional nonanimated biofeedback, with both arms receiving background urotherapy (Figure 3).

In the studies comparing animated biofeedback plus urotherapy with urotherapy alone, the direction of effect generally favored the animated biofeedback groups, suggesting that biofeedback-based pelvic floor retraining may improve bladder emptying when added to conservative management. However, this comparison reflects the combined effect of biofeedback and animation, rather than the independent effect of gamification itself.

In contrast, the study comparing animated biofeedback directly with nonanimated biofeedback did not demonstrate a clear advantage of the animated interface. Both treatment arms showed clinical improvement, indicating that the principal therapeutic effect may derive from biofeedback-mediated neuromuscular retraining rather than animation alone.

The exploratory random-effects meta-analysis showed no statistically significant overall reduction in PVR with animated or gamified biofeedback compared with control regimens. The pooled MD was -6.83 mL (95% CI, -29.27 to 15.62; p = 0.55), with substantial heterogeneity (I² = 89%). The wide CI indicates considerable imprecision, and the true effect may range from a clinically meaningful benefit to minimal or no benefit. Given the limited number of studies, differences in diagnosis, and heterogeneity in comparator groups, this pooled estimate should be interpreted with caution and not considered confirmatory evidence of superiority.

The clinical relevance of the observed MD is also uncertain. A reduction of approximately 7 mL in PVR may have different implications depending on patient age, bladder capacity, baseline residual volume, and underlying diagnosis. Because the included studies enrolled children across a wide age range and did not apply uniform age-adjusted thresholds for abnormal PVR, the pooled absolute difference cannot be directly translated into a universal clinically meaningful benefit.

Uroflowmetry Parameters

Uroflowmetry-related outcomes were reported descriptively across the included studies, but the parameters and reporting formats were not sufficiently consistent to permit quantitative pooling. Reported variables included maximum urinary flow rate, voiding time, uroflow pattern normalization, voided volume, and EMG activity during voiding.

In the studies comparing animated biofeedback plus urotherapy with urotherapy alone, improvements in uroflowmetry parameters were generally more apparent in the animated biofeedback groups. These improvements included better voiding coordination, reduction in abnormal pelvic floor activity during voiding, shorter voiding time, and more favorable urinary flow patterns. These findings support the physiological rationale that biofeedback may help children learn pelvic floor relaxation during micturition.

However, the study comparing animated with nonanimated biofeedback did not show a clear superiority of the animated interface. Both groups demonstrated improvement after treatment, suggesting that the biofeedback process itself may be the primary driver of uroflowmetry improvement. Therefore, while uroflowmetry findings were directionally supportive of biofeedback-based therapy, they do not provide robust evidence that animation or gamification independently improves voiding dynamics beyond conventional biofeedback.

Because uroflowmetry outcomes were heterogeneous in their definitions, timing, and statistical reporting, these findings should be interpreted as supportive descriptive evidence rather than as pooled comparative evidence.

Symptom Scores and Clinical Success

Symptom scores and clinical success were reported across the included studies, but definitions varied substantially and were therefore not suitable for quantitative synthesis. Clinical response was defined differently across studies, including reductions in voiding symptom scores, improvements in continence or voiding frequency, normalization of uroflowmetry or EMG findings, reductions in PVR urine volume, or combined subjective and objective improvements.

In the studies comparing animated biofeedback plus urotherapy with urotherapy alone, symptom improvement appeared more favorable in the animated biofeedback groups. These studies reported reductions in voiding symptoms and improvements in functional parameters, suggesting that biofeedback-based pelvic floor retraining may provide additional benefit for children who require more structured neuromuscular re-education than urotherapy alone can provide.

However, this finding should not be interpreted as evidence that animation itself is superior. In the study directly comparing animated and nonanimated biofeedback, clinical success rates were broadly comparable between the groups. This suggests that the therapeutic effect may be primarily attributable to the biofeedback intervention rather than to the animated or gamified interface. The role of animation may therefore be more related to child engagement, motivation, and treatment participation than to a clearly demonstrated independent physiological effect.

Because treatment success was not standardized across studies and was based on mixed combinations of subjective symptoms and objective findings, the available evidence does not support a pooled estimate for this outcome. Symptom and clinical success findings should therefore be interpreted narratively and in relation to the specific comparator used in each study.

Summary of Quantitative Findings

The exploratory quantitative synthesis did not demonstrate a statistically significant overall advantage of animated or gamified biofeedback for reducing PVR urine volume. Although the pooled estimate numerically favored animated biofeedback, the CI was wide and crossed the null effect, indicating substantial imprecision. In addition, heterogeneity was high, reflecting differences in diagnosis, baseline bladder emptying dysfunction, treatment protocols, follow-up duration, and comparator structure.

The direction of effect appeared more favorable in studies comparing animated biofeedback plus urotherapy with urotherapy alone. However, this comparison does not isolate the specific contribution of animation because the intervention groups also received biofeedback-based pelvic floor retraining. Conversely, the study comparing animated with nonanimated biofeedback did not show a clear incremental benefit of animation. Therefore, the available evidence suggests that biofeedback may be beneficial as a neuromuscular retraining strategy, but it remains uncertain whether animation or gamification provides additional therapeutic value beyond conventional biofeedback.

Overall, the quantitative findings should be interpreted as exploratory and hypothesis-generating rather than confirmatory. The small number of studies, wide CI, and substantial heterogeneity limit the strength of inference that can be drawn from the pooled estimate. Future studies using standardized diagnostic criteria, age-adjusted PVR thresholds, consistent comparator groups, and harmonized outcome definitions are needed before a definitive conclusion can be made regarding the independent clinical value of animated or gamified biofeedback in pediatric functional voiding dysfunction.

Certainty of Evidence

The certainty of evidence for the exploratory PVR outcome was considered low. This judgment was mainly driven by the very small number of included studies, limited total sample size, substantial statistical heterogeneity, and imprecision of the pooled estimate. Although most included studies had acceptable risk-of-bias profiles, the evidence was weakened by indirectness due to differences in diagnoses and comparator structures. Specifically, the available studies did not address a single uniform clinical question because some evaluated the addition of animated biofeedback to urotherapy, whereas others evaluated animation as an interface modification compared with nonanimated biofeedback.

Publication bias could not be formally assessed because fewer than 10 studies were included. Therefore, no firm conclusion can be drawn regarding small-study effects or selective publication. Overall, the available evidence should be viewed as preliminary and hypothesis-generating, supporting the need for larger comparative trials rather than establishing definitive superiority of animated or gamified biofeedback.

Discussion

This systematic review and meta-analysis evaluated the effectiveness of animated or gamified pelvic floor biofeedback in children with functional voiding dysfunction, focusing exclusively on PVR urine volume (PVR) as the pooled quantitative outcome. Although animated biofeedback demonstrated consistent within-study improvements in bladder emptying parameters, the pooled analysis did not show a statistically significant overall reduction in PVR compared with control regimens. Importantly, substantial heterogeneity was observed, indicating that the effect of animation may be context-dependent rather than universally superior.

From a physiological perspective, PVR represents a direct marker of voiding efficiency and detrusor-sphincter coordination [1,2]. In DV, persistent pelvic floor contraction during micturition increases outlet resistance, leading to incomplete bladder emptying [2]. Similarly, in UAB, impaired detrusor contractility results in prolonged voiding and residual urine accumulation [3]. Biofeedback aims to retrain pelvic floor relaxation and restore coordinated voiding mechanics [5]. Therefore, improvement in PVR reflects successful neuromuscular reeducation rather than merely subjective symptom relief.

In the included trials comparing animated biofeedback with standard urotherapy alone, the direction of effect consistently favored the animated intervention. This aligns with prior evidence suggesting that biofeedback enhances outcomes beyond conservative management in children refractory to urotherapy [5,11]. The mechanism is plausible: urotherapy addresses behavioral components, whereas biofeedback directly targets neuromuscular coordination through real-time EMG visualization. Animated interfaces may further reinforce motor learning by sustaining attention and facilitating repetition of correct voiding patterns [6,7]. Specifically, one of the earliest randomized trials in children with dysfunctional elimination syndrome demonstrated that game-based animated biofeedback combined with pelvic floor exercises produced significant reductions in voiding dysfunction symptom scores and PVR volume, with normalization of uroflowmetry patterns maintained at both 6- and 12-month follow-ups [10].

However, when animated biofeedback was compared directly with nonanimated biofeedback, no statistically significant difference in clinical success or objective parameters was observed [9]. This finding suggests that the therapeutic benefit primarily derives from biofeedback itself rather than the animated interface. Animation may improve engagement and cooperation, particularly in younger children, but this does not necessarily translate into measurable superiority in objective urodynamic parameters. This distinction is clinically relevant, especially in resource-limited settings where simpler biofeedback systems may be more accessible.

The substantial heterogeneity (I² = 89%) observed in the pooled analysis warrants careful interpretation. Differences in underlying diagnoses (DV versus UAB), baseline PVR values, treatment frequency, session number, and comparator structure likely contributed to variability. Notably, UAB and DV, while both functional disorders, involve distinct pathophysiological mechanisms [3]. Therefore, pooling across these entities may dilute intervention effects specific to each condition. Additionally, studies comparing animated biofeedback to urotherapy alone are methodologically different from those comparing animated to non-animated biofeedback, further amplifying heterogeneity.

Previous systematic reviews evaluating biofeedback in pediatric nonneuropathic voiding disorders did not isolate animated or gamified systems as a distinct intervention category [7]. As digital and gamified rehabilitation tools become increasingly integrated into pediatric practice, understanding whether animation confers incremental clinical value is essential. Although our quantitative synthesis did not demonstrate statistical superiority for PVR, narrative findings suggest that animated biofeedback may offer meaningful benefits when compared with urotherapy alone, particularly in children with persistent abnormal voiding patterns [8].

Beyond physiological outcomes, engagement and adherence represent critical determinants of therapeutic success in pediatric populations [4]. Gamified interfaces may enhance intrinsic motivation and reduce dropout rates, even if hard urodynamic endpoints are comparable [12]. Emerging literature in pediatric rehabilitation suggests that interactive digital platforms improve participation and motor learning retention [13]. Thus, while animation may not independently alter PVR magnitude across all settings, it may indirectly influence long-term behavioral maintenance and the prevention of recurrence [13-16].

Another important limitation relates to the interpretation of PVR urine volume as a clinical endpoint. Although PVR is an objective and clinically relevant marker of bladder emptying, the clinical meaning of a small absolute difference in PVR is not uniform across pediatric patients. In the present analysis, the pooled MD was -6.83 mL, but the 95% CI was wide and crossed the null effect. This means that the true effect may range from a potentially meaningful reduction to little or no benefit. Moreover, a difference of several milliliters may have different clinical implications depending on baseline bladder capacity, age, voided volume, and initial severity of residual urine retention. Therefore, statistical interpretation alone is insufficient, and the pooled estimate should be viewed in the context of age-adjusted bladder function and individual clinical presentation.

The wide age range of included participants further limits direct clinical interpretation. Children between 5 and 16 years differ substantially in expected bladder capacity, voiding habits, pelvic floor control, ability to cooperate with biofeedback training, and normal thresholds for residual urine. Younger children may benefit more from animated interfaces because visual reinforcement can improve attention and engagement, whereas older children may respond adequately to conventional verbal or visual biofeedback without gamification. However, the included studies did not provide sufficient stratified data by age group to determine whether treatment response differed between younger and older children. Future studies should report age-adjusted PVR values or stratified outcomes to clarify whether animated biofeedback has greater value in specific developmental stages.

Potential sex-related differences also deserve consideration. Pediatric functional voiding disorders may differ between boys and girls in symptom presentation, pelvic floor behavior, urinary tract infection risk, and response to conservative therapy. Girls may present more frequently with recurrent urinary tract infections and residual urine-related symptoms, whereas boys may have different voiding patterns and anatomical considerations that influence uroflowmetry interpretation. The included studies did not consistently report treatment effects stratified by sex, preventing assessment of whether animated or gamified biofeedback has differential effectiveness between male and female patients. This represents an important gap because sex distribution may influence both baseline risk and treatment response.

The current findings also highlight the need to distinguish between the value of biofeedback and the value of animation. If animated biofeedback is compared with urotherapy alone, any observed benefit may simply reflect the established therapeutic role of pelvic floor biofeedback rather than the gamified interface. In contrast, direct comparison with nonanimated biofeedback is necessary to evaluate whether animation provides additional clinical benefit. Based on the available evidence, the latter question remains unanswered. Therefore, the conclusion should not be that animated biofeedback is superior, but rather that the evidence is insufficient to confirm whether animation adds measurable benefit beyond conventional biofeedback.

The small number of included studies also limits the appropriateness and interpretability of formal meta-analysis. With only three studies and substantial heterogeneity, the pooled estimate is statistically unstable and highly dependent on study composition. Formal subgroup analysis by diagnosis or comparator type was not feasible, and assessment of publication bias was not performed because funnel plot interpretation is unreliable with fewer than ten studies. For this reason, the quantitative synthesis in this review should be regarded as exploratory and hypothesis-generating. The narrative synthesis may provide a more clinically meaningful interpretation than the pooled estimate alone.

From a methodological perspective, future research should avoid combining distinct comparator frameworks within a single undifferentiated analysis. Trials evaluating animated biofeedback as an adjunct to urotherapy should be analyzed separately from trials comparing animated with nonanimated biofeedback. Similarly, DV and nonneuropathic UAB should ideally be studied or reported separately because their mechanisms and expected treatment responses are not identical. Standardization of diagnostic criteria, treatment protocols, follow-up intervals, and outcome definitions would substantially improve the comparability of future studies.

Finally, future studies should evaluate outcomes beyond PVR alone. Although PVR is objective, it does not fully capture symptom burden, quality of life, urinary tract infection frequency, recurrence, treatment adherence, or child engagement. These outcomes are particularly relevant when assessing gamified interventions because the main theoretical advantage of animation may be improved motivation and participation rather than direct physiological superiority. Therefore, future randomized trials should include both objective urodynamic parameters and patient-centered outcomes to determine whether animated interfaces provide meaningful practical benefit in real-world pediatric urology care.

Future research should focus on multicenter randomized trials with standardized outcome definitions and adequate power to detect clinically meaningful differences. Stratified analyses by diagnostic subgroup (DV vs. UAB) would clarify whether certain phenotypes derive greater benefit from animated platforms. Additionally, the incorporation of patient-reported outcome measures and adherence metrics may provide a more comprehensive understanding of animation’s role in pediatric voiding rehabilitation. A longer follow-up is also essential to evaluate recurrence rates and the sustainability of neuromuscular retraining.

## Conclusions

This systematic review with exploratory meta-analysis found that the available evidence does not demonstrate a statistically significant overall advantage of animated or gamified biofeedback in reducing PVR among children with functional voiding dysfunction. Although some individual studies suggested favorable outcomes when animated biofeedback was added to standard urotherapy, this comparison does not isolate the independent effect of animation because biofeedback itself was also introduced as part of the intervention. In the available direct comparison between animated and nonanimated biofeedback, no clear additional benefit of the animated interface was demonstrated.

Therefore, current evidence is insufficient to conclude that animation or gamification provides a definitive therapeutic advantage beyond conventional biofeedback. The pooled estimate should be interpreted cautiously because of the small number of studies, substantial clinical and statistical heterogeneity, differences in diagnosis and comparator structure, and imprecision of the effect estimate. Animated biofeedback may remain useful as an engagement-enhancing tool in selected pediatric patients, but its independent clinical value requires confirmation in larger, well-designed comparative studies with standardized diagnostic criteria, age-adjusted PVR interpretation, consistent outcome definitions, and longer follow-up.
